# The Possible Contribution of P-Glycoprotein in the Protective Effect of Paeonol against Methotrexate-Induced Testicular Injury in Rats

**DOI:** 10.3390/ph13090223

**Published:** 2020-08-29

**Authors:** Mohamed A. Morsy, Asmaa M. Abdel-Aziz, Sara M. N. Abdel-Hafez, Katharigatta N. Venugopala, Anroop B. Nair, Seham A. Abdel-Gaber

**Affiliations:** 1 Department of Pharmaceutical Sciences, College of Clinical Pharmacy, King Faisal University, Al-Ahsa 31982, Saudi Arabia; kvenugopala@kfu.edu.sa (K.N.V.); anair@kfu.edu.sa (A.B.N.); 2 Department of Pharmacology, Faculty of Medicine, Minia University, El-Minia 61511, Egypt; asmaaabdelaziz303@yahoo.com (A.M.A.-A.); sehamabdelwakeel@yahoo.com (S.A.A.-G.); 3 Department of Histology and Cell Biology, Faculty of Medicine, Minia University, El-Minia 61511, Egypt; sara_histology@yahoo.com; 4 Department of Biotechnology and Food Technology, Durban University of Technology, Durban 4001, South Africa

**Keywords:** paeonol, methotrexate, P-glycoprotein, testicular injury, oxidative stress, tumor necrosis factor-α, caspase 3

## Abstract

Paeonol, a phenolic ingredient in the genus *Paeonia*, possesses antioxidant and anti-inflammatory effects. Methotrexate (MTX) is a commonly used chemotherapeutic agent; however, its germ cell damage is a critical problem. P-glycoprotein (P-gp), an efflux transporter, is a member of the blood–testis barrier. The present study evaluated the protective effect of paeonol on MTX-induced testicular injury in rats with the exploration of its mechanism and the possible contribution of P-gp in such protection. Testicular weight, serum testosterone, and testicular P-gp levels were measured. Testicular oxidant/antioxidant status was evaluated via determining the levels of malondialdehyde, total nitrite, reduced glutathione, and superoxide dismutase activity. The inflammatory cytokine tumor necrosis factor-alpha (TNF-α) and the apoptotic marker caspase 3 were estimated immunohistochemically. Testicular histopathology and spermatogenesis scores were also examined. MTX caused histopathologically evident testicular damage with decreased testicular weight, testosterone level, and spermatogenesis score, as well as significant increases in oxidative, inflammatory, and apoptotic responses. Paeonol significantly restored testicular weight, testosterone level, spermatogenesis score, and oxidant/antioxidant balance. Moreover, paeonol increased the testicular P-gp level and significantly decreased TNF-α and caspase 3 immunostaining. In conclusion, paeonol offered a protective effect against MTX-induced testicular injury through its antioxidant, anti-inflammatory, and antiapoptotic effects, as well as by increasing testicular P-gp level.

## 1. Introduction

Methotrexate (MTX) is a chemotherapeutic agent that has been used in clinical practice since the 1950s. MTX is a folic acid antagonist, which is used to treat various types of malignancies and autoimmune diseases [1]. Inhibition of dihydrofolate reductase by MTX results in suppression of purine and pyrimidine nucleotide production, and subsequently, inhibition of DNA and RNA synthesis, especially in rapidly dividing malignant cells [1,2]. Unfortunately, the cytotoxic effects of MTX are not limited to malignant cells but can also affect normal cells in different organs [3,4,5], including the testis [6,7]. Increased oxidative stress, inflammation, and apoptosis may also account for MTX-induced cellular damage [5,6]. However, the exact mechanisms responsible for MTX-induced testicular damage are yet to be fully elucidated. The blood–testis barrier divides the seminiferous epithelium anatomically into basal and apical compartments; thus, creating a distinctive microenvironment for spermatogenesis in the apical compartment and prohibiting the accumulation of harmful substances in the testes, and functionally protecting against the destruction of spermatozoa by the immune response [8]. Besides the disruption of the blood–testis barrier, MTX can affect the reproductive capacity via suppression of the cellular proliferation of germinal and non-germinal (Sertoli and Leydig cells) elements of the seminiferous tubules [9].

The 175 kDa P-glycoprotein (P-gp) is the ATP-binding cassette (ABC) subfamily B member 1 (ABCB1) efflux transporter that was first identified in tumor cells showing resistance to a wide range of chemotherapeutic drugs, including MTX [10]. Later, the expression of functional P-gp was verified in several normal cells, including Sertoli cells that largely create the blood–testis barrier. Importantly, the presence of a functionally adequate blood–testis barrier is broadly dependent on the ability of efflux pump transporters such as P-gp to limit testicular drug and toxicant accumulation [11,12]. Indeed, the work of Droździk et al. introduced further evidence supporting the P-gp-mediated testicular protection hypothesis by unravelling a positive association between a single nucleotide (3435C>T) polymorphism in the ABCB1 gene and male infertility [13]. Therefore, the upregulation of P-gp expression and/or activity is considered a promising and effective cytoprotective mechanism against testicular chemical insults [14].

Paeonol, a phenolic active constituent isolated from the genus *Paeonia*, possesses remarkable antioxidant and anti-inflammatory effects, which are beneficial in many diseases, such as gastric ulcer [15], hypertension [16], arthritis [17], and epilepsy [18]. Recently, we have demonstrated that paeonol can also protect against testicular ischemia-reperfusion injury [19]. On the other hand, the expression and/or activity of P-gp can be modulated by numerous natural products [20]; meanwhile, P-gp accepts paeonol as a substrate [21]. To the best of our knowledge, previous studies neither evaluated the protective effect of paeonol against MTX-induced testicular injury nor described the role of P-gp in paeonol protection against any testicular injury model. The aim of the current study, therefore, was to evaluate the possible protective effects of paeonol against MTX-induced testicular injury and to explore the various mechanisms that underlie these protective effects, including the potential effect on testicular P-gp expression as a plausible mechanism.

## 2. Results

### 2.1. Effect of Paeonol on Testicular Weight and Serum Testosterone Level

The administration of MTX significantly decreased the rat testicular weight compared to normal control rats. On the other hand, treatment with paeonol significantly increased the testicular weight in comparison with MTX-challenged rats. The paeonol alone group showed no significant difference in the testicular weight compared to the control group. Besides, the serum testosterone level significantly decreased in MTX-intoxicated rats, while paeonol treatment significantly increased the testosterone level compared to the MTX group (Table 1).

### 2.2. Effect of Paeonol on Testicular P-gp Level

The level of testicular P-gp increased, albeit insignificantly, in the MTX-challenged group compared to the normal control rats. On the other hand, paeonol treatment resulted in significantly higher levels of the P-gp in the MTX-challenged rats when compared to the MTX-treated group alone (Table 1).

### 2.3. Effect of Paeonol on Histopathological Changes and Spermatogenesis Scoring

**Figure 1:**
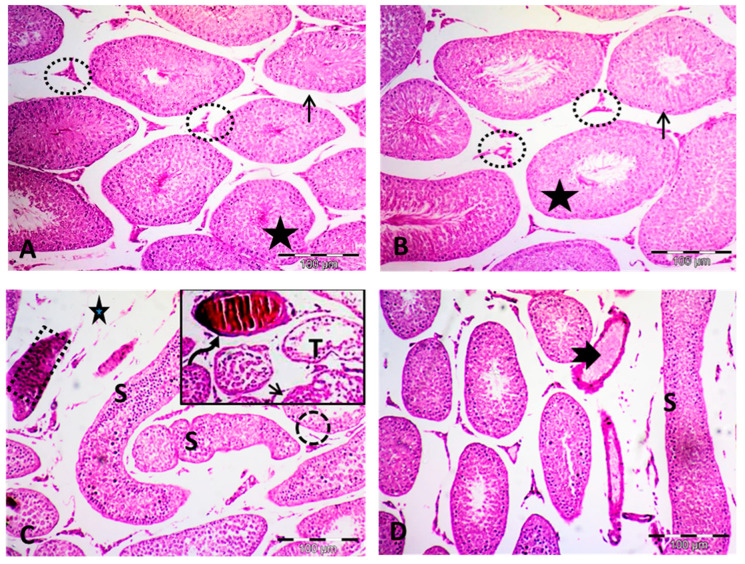
Photomicrographs of the testis (hematoxylin and eosin ×100). (**A**,**B**) Control and paeonol groups respectively, showing the same histological picture. The seminiferous tubules (stars) are seen lined with germinal epithelium. The interstitial cells of Leydig (circles) are observed between these tubules. Notice the sperms occupying the lumen of seminiferous tubules. The tubules are seen laying on the basement membrane (arrows). (**C**) MTX group showing several distorted seminiferous tubules (S) lined by darkly stained acidophilic cells (rectangle) with dense nuclei. Notice the focal reduction of the germinal cells (circle). A widened interstitium (star) is also noticed. The insert shows some tubules (T) that have nearly lost their germinal epithelium lining. The separated basement membrane (short arrow) can also be seen. Dilated congested blood vessels (curved arrow) occupying the interstitium are also noticed. (**D**) Paeonol + MTX group showing apparent normalization of the seminiferous tubules, but still few tubules appear with some distortion (S). Less widening of blood vessels (notched arrow) are seen.

Analysis of the hematoxylin and eosin (H&E)-stained testis sections of both the control and the paeonol-treated groups showed normal histological architecture. The seminiferous tubules were seen lined with germinal epithelium. Leydig cells were observed between these tubules. Spermatozoa occupying the lumen of the seminiferous tubules were also detected. The tubules were seen lying on intact basement membranes (Figure 1A,B), with an appropriate height of germinal epithelial lining and normal histopathological grading (Table 2).

Regarding the MTX group, various morphological changes were noticed, including several distorted seminiferous tubules with variable shapes. Some of these tubules are lined by darkly stained cytoplasm and nuclei. Focal reduction of the germinal cells was frequently seen with a significant decrease in the height of the epithelial germinal layer. Moreover, in some sections, the seminiferous tubules nearly lost their germinal cell lining. Focal separated basement membranes were also seen. Dilated congested blood vessels occupying widened interstitium were clearly noticed in this group. A significant elevation of Cosentino score of histopathological injury was also seen in this MTX-treated group (Figure 1C and Table 2). Animals challenged with MTX showed significantly decreased spermatogenesis, as confirmed by the application of the Johnsen scoring system. Alternatively, paeonol + MTX-treated rats showed significantly improved spermatogenesis compared to the MTX-treated group (Table 2).

Paeonol + MTX-treated rats showed amelioration of almost all morphological abnormalities observed in the MTX-treated rats, with restoration of the germinal epithelial lining height and decreased Cosentino histopathological injury score. Although the testicular sections of this group showed apparent preservation of the seminiferous tubules, a few tubules appeared with some distortion. Less widening of blood vessels was also seen (Figure 1D and Table 2).

### 2.4. Effect of Paeonol on Oxidative Stress Parameters

Testicular malondialdehyde (MDA) and total nitrite (NOx) levels were elevated; meanwhile, the level of reduced glutathione (GSH), as well as the activity of superoxide dismutase (SOD), were decreased in the MTX-challenged rats in comparison with the normal control rats. Pretreatment with paeonol before the MTX challenge significantly decreased the levels of both MDA and NOx and increased the GSH level and SOD activity compared to the MTX-treated group. The paeonol alone treatment did not significantly alter the above-mentioned parameters in comparison with values observed in the normal control group (Table 3).

### 2.5. Effect of Paeonol on Tumor Necrosis Factor-Alpha (TNF-α) and Caspase 3 Immunostaining

**Figure 2:**
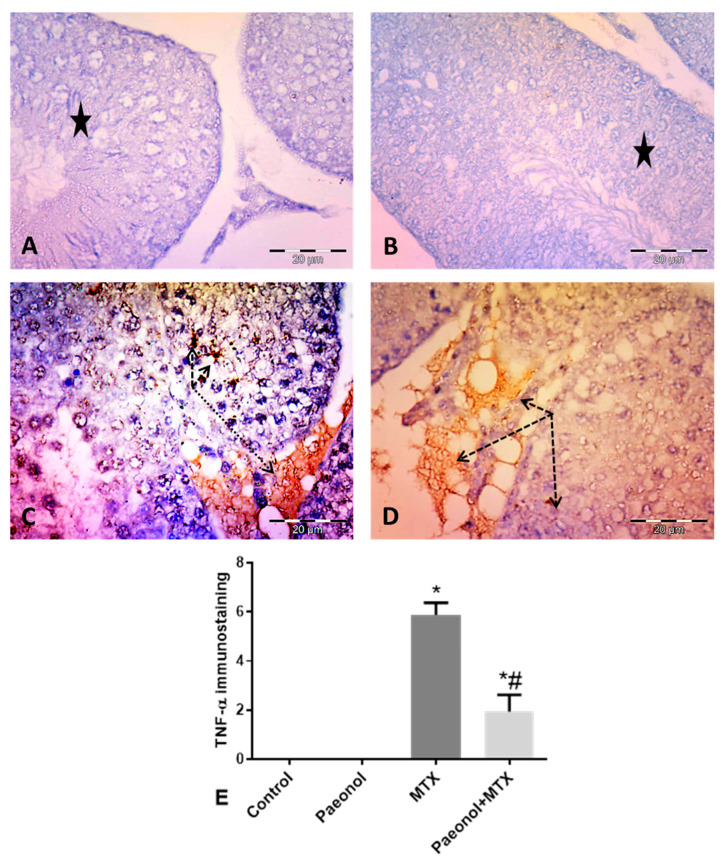
Immunohistochemical expression of testicular tumor necrosis factor-alpha (TNF-α) (×400). (**A**,**B**) Control and paeonol groups respectively, showing negative TNF-α immunoreactivity (stars) in the germinal cells. (**C**) MTX group showing cytoplasmic TNF-α immunoreactivity in the germinal cells and in the interstitial cells (dashed arrows). (**D**) Paeonol + MTX group showing faint cytoplasmic TNF-α expression in the germinal and interstitial cells (dashed arrows). (**E**) Mean area fraction of TNF-α immunostaining. Results are mean ± SEM (*n* = 8). *^,#^ Significantly different (*p* < 0.05) from control and MTX groups, respectively. MTX: methotrexate.

**Figure 3:**
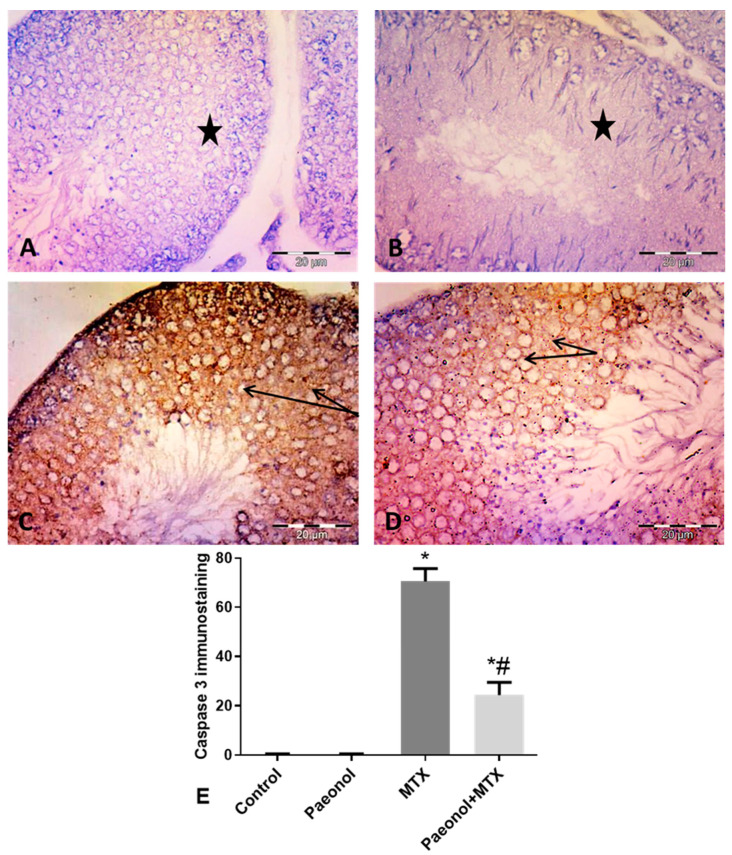
Immunohistochemical expression of testicular caspase 3 (×400). (**A**,**B**) Control and paeonol groups respectively, showing negative caspase 3 immunoreactivity (stars) in the germinal cells. (**C**) MTX group showing positive diffuse intense cytoplasmic caspase 3 immunoreactivity (arrows) in the germinal cells. (**D**) Paeonol + MTX group showing less cytoplasmic caspase 3 immunoreactivity (arrows) in germinal cells. (**E**) Mean area fraction of caspase 3 immunostaining. Results are mean ± SEM (*n* = 8). *^,#^ Significantly different (*p* < 0.05) from control and MTX groups, respectively. MTX: methotrexate.

Immunohistochemical staining of testicular tissues for the detection of TNF-α and caspase 3 revealed negative immunoreactivity in the germinal cells of the control and paeonol-treated groups (Figure 2A,B and Figure 3A,B, respectively). The MTX-intoxicated group showed high cytoplasmic immunoreactivity for both parameters in the germinal and interstitial cells (Figure 2C and Figure 3C, respectively). On the other hand, tissues of the paeonol + MTX-treated group showed lower cytoplasmic expression levels of both TNF-α and caspase 3 in these cells in comparison with the MTX-treated group (Figure 2D and Figure 3D, respectively). There was a significant increase in the TNF-α and caspase 3 area fraction in the MTX-treated group in comparison with the control group, while there was a significant decrease in the area fraction of both parameters in the paeonol + MTX-treated group compared to the MTX-treated group (Figure 2E and Figure 3E, respectively).

## 3. Discussion

Testicular injury is a serious adverse effect of MTX therapy, thus the preservation of germinal cells is an essential target during cancer chemotherapy [6,7]. In line with previous reports [22,23], MTX treatment in the current study was associated with a significant decrease in testicular weight, indicative of testicular atrophy. Pretreatment with paeonol, however, prevented the MTX-induced testicular atrophy, as shown by preservation of testicular weight. The current results demonstrated severe histopathological testicular distortion of the normal architecture in the MTX-challenged rats, evident by heightened Cosentino histopathological injury scores and reduced height of the germinal epithelium lining. Moreover, MTX resulted in testicular functional impairment in the form of significantly decreased serum testosterone level and impaired spermatogenesis. These results are supported by the findings of previous studies [23,24,25], which demonstrated that a single dose of MTX was associated with testicular structural damage, impaired spermatogenesis, and decreased testosterone. Alternatively, pretreatment with paeonol mitigated the MTX-induced testicular injury, with marked preservation of normal testicular architecture as well as the functional ability of the treated testes, which is in agreement with our recent findings showing protection by paeonol against testicular ischemia-reperfusion injury [19].

P-gp is an efflux transporter, which physiologically regulates substrate passage into and accumulation in different organs, including the testis; thus, it can remarkably diminish intracellular drug or toxicant concentrations [11]. In the current study, a single dose of MTX resulted in an insignificant effect on P-gp protein level compared to normal control rats. The effect of MTX on P-gp expression is controversial. For example, Norris et al. [26] reported that MTX led to increased expression of P-gp in CEM/MTX R cells. On the other hand, MTX resulted in decreased rat testicular P-gp level [23]. This probably means that there is a differential response of P-gp to MTX treatment in non-cancerous versus cancerous cells. Paeonol, in the present study, significantly increased the P-gp protein level compared to MTX-challenged rats, and thus can protect against MTX-induced testicular injury via efflux of MTX out of the testis. It is known that P-gp accepts MTX [10] and paeonol [21] as substrates, so testicular herb–drug interactions are presumed. Interestingly, and contrary to our results, paeonol was reported to have the ability to reverse paclitaxel resistance in MCF-7/PTX cells by downregulation of P-gp expression [27]. Accordingly, whether paeonol protection against MTX-induced testicular toxicity would disturb the anticancer efficacy of MTX is the question that has arisen. However, we previously showed that resveratrol, a natural phenol, has the same dual beneficial effect, as it potentiated MTX cytotoxicity in prostate cancer cells while protecting against testicular injury through the upregulation of testicular Mrp3, another ABC efflux transporter [24]. Nevertheless, further studies are required to detect the level of P-gp in non-malignant versus malignant cells exposed to MTX and treated with paeonol, as well as to know whether or not paeonol-induced P-gp upregulation is enough to confer MTX-resistance, which is indeed multifactorial [26].

Oxidative stress, which arises as a result of the imbalance between reactive oxygen species (ROS) and the endogenous antioxidant system, is an important factor in MTX-induced testicular injury [6,7,22,25]. MTX can increase ROS production by the elevation of homocysteine [28], reduction of NADPH [29], and activation of NADPH oxidase [30]. In the current study, increased oxidative stress was implicated in the pathogenesis of testicular injury in response to MTX administration, evident by increased testicular pro-oxidant MDA and nitric oxide (NO) levels together with decreased testicular antioxidant GSH level and SOD activity. Compatible with these results, several previous reports showed similar findings [6,25,31]. The lipid peroxidation product MDA, which harmfully affects sperm quality [32], is a well-known marker of oxidative stress. Besides, high levels of the free radical gaso-transmitter, NO, which mediates lipid peroxidation and proapoptotic effects [33] and decreases intracellular GSH [34], can cause impairment of spermatogenesis [35]. On the other hand, the nonenzymatic antioxidant GSH plays a distinctive role in the protection against oxidative stress via scavenging of the ROS, working as a cofactor for antioxidant enzymes, as well as facilitating the detoxification of xenobiotics [36]. Also, SOD, which is an effective first-line defense mechanism against oxidative stress, catalyzes the dismutation of the deleterious superoxide radical to less noxious products [37]. In the present study, paeonol administration protected the testis against the MTX-induced increase of oxidative stress. The paeonol + MTX-treated rats demonstrated lower levels of testicular lipid peroxidation and NO, as well as enhanced GSH levels and SOD activity, which is in agreement with previous reports [5,15,18,19]. The suppressive effect of paeonol on lipid peroxidation might be due to its inherent antioxidant activity [15,19] and decreasing NO production [5,38].

The inflammatory cytokine TNF-α plays a significant role in the pathogenesis of MTX-induced testicular injury [22,23,24,25]. TNF-α induces oxidative stress, the release of other inflammatory mediators, NO production, and apoptosis [39,40,41], leading to impairment of spermatogenesis [35] and a decrease in testosterone level [42]. In harmony with the present study, previous reports demonstrated similar findings regarding the ability of paeonol to attenuate the testicular increase in MTX-induced TNF-α expression [5,19,43]. On the other hand, caspase 3, a key enzyme in programmed cell death pathways [44], is implicated in germ cell apoptosis during MTX-induced testicular injury [45], which is consistent with the results of the current study. Paeonol, in the present study, decreased the MTX-induced elevation in caspase 3 immunostaining, which is supported by previous reports [5,15]. Since the interplay between inflammation and increased oxidative stress is a well-established cell death signal [46], the decreased expression of caspase 3 in response to paeonol treatment may be secondary to its antioxidant and anti-inflammatory activities. Remarkably, in contrast to the present data, paeonol was reported to exert anticancer activity by induction of apoptosis via activation of caspase 3 [47]. Such contradictory effect of paeonol on caspase 3 expression, hence the regulation of apoptosis, may be ascribed to different signaling mechanisms in play in normal versus cancer cells.

## 4. Materials and Methods

### 4.1. Animals

Twenty-four male Wistar rats weighing 200 ± 20 g were purchased from the National Research Center (Giza, Egypt) and were kept at standard housing conditions (24 °C, 45% humidity, and 12 h light/dark cycle). Rats were supplied with the ordinary chew and tap water ad libitum and were left to acclimatize for 7 days before inclusion in the experiment. The current study was approved (630/6/2020) and performed following the research ethical standards of the Faculty of Medicine-Research Ethics Committee, “FMREC”, Minia University, Egypt, in compliance with EU directive 2010/63/EU for care and utilization of laboratory animals.

### 4.2. Drugs, Chemicals, and Antibodies

Paeonol and MTX were procured from Sigma-Aldrich (St. Louis, MO, USA) and Minapharm Pharmaceuticals (Cairo, Egypt), respectively. Paeonol was prepared in 0.5% carboxymethyl cellulose (CMC) solution. Ready-to-use TNF-α and caspase 3 rabbit polyclonal antibodies were purchased from Thermo Fisher Scientific (Waltham, MA, USA). All other used chemicals were for analytical purposes and were obtained from their commercial sources.

### 4.3. Experimental Design

Rats were randomly allocated into four groups, of six animals each. The control group received the vehicles (single intraperitoneal (i.p.) injection of saline and an oral daily dose of CMC for 10 days, beginning 5 days before saline administration). The paeonol alone group received a single i.p. injection of saline and an oral daily dose of paeonol (100 mg/kg/day) [5] suspended in CMC for 10 days, beginning 5 days before saline administration. The MTX group received a single i.p. injection of MTX (20 mg/kg) [5] and an oral daily dose of CMC for 10 days, beginning 5 days before MTX administration. The paeonol + MTX group received a single i.p. injection of MTX (20 mg/kg) and an oral daily dose of paeonol (100 mg/kg/day) for 10 days, beginning 5 days before MTX administration.

After 5 days of MTX injection, rats were euthanized and blood was withdrawn via a left ventricular prick. The blood was centrifuged at 5000 rpm for 15 min for the determination of serum testosterone level. Testes were dissected, washed by normal saline, and weighed. One testis was fixed in Bouin’s solution for histopathological and immunohistochemical examinations and the other testis was homogenized in 20% *w/v* ice-cold phosphate buffer (pH 7.4). The homogenate was centrifuged at 4000 rpm for 15 min and the supernatant was used for testicular biochemical measurements.

### 4.4. Biochemical Analysis

#### 4.4.1. Determination of Serum Testosterone Level

Serum testosterone level was measured via a testosterone enzyme-linked immunosorbent assay (ELISA) kit (Cayman Chemical, Ann Arbor, MI, USA) according to the manufacturer’s instructions.

#### 4.4.2. Determination of Testicular P-gp Level

Testicular P-gp level was evaluated using a P-gp ELISA kit (US Biological, Salem, MA, USA) according to the manufacturer’s instructions.

#### 4.4.3. Determination of Testicular Oxidative Stress Biomarkers

To assess the oxidative damage induced by MTX, testicular lipid peroxidation was determined as a thiobarbituric acid-reacting substance and expressed as equivalents of MDA, using 1,1,3,3-tetramethoxypropane as a standard [48], and results were expressed as nmol/g tissue. NO content was determined as NOx, which was measured after the reduction of nitrate to nitrite by copperized cadmium granules in glycine buffer at pH 9.7. Quantitation of nitrite was based on the Griess reaction, in which a chromophore with a strong absorbance detected at 540 nm is formed by a reaction of nitrite with a mixture of naphthyl ethylenediamine and sulfanilamide [49], and results were expressed as nmol/g tissue. GSH level was chemically assessed using a method described by Moron et al. [50]. The method is based on the fact that the sulfhydryl group of GSH interacts with 5,5′-dithiobis(2-nitrobenzoic acid) (Ellman’s reagent) that produce a yellow-colored 5-thio-2-nitrobenzoic acid, which was measured spectrophotometrically at 412 nm, and results were expressed as nmol/g tissue. SOD enzyme activity was also chemically assessed via a previously described method [51], which depends on the fact that SOD inhibits pyrogallol autoxidation. One unit of SOD is equal to the enzyme level which can inhibit 50% of pyrogallol autoxidation. SOD activity was detected colorimetrically at 420 nm and results were expressed as U/g tissue.

### 4.5. Histopathological and Immunohistochemical Examinations

One testis from each animal was fixed in Bouin’s solution, dehydrated in ascending degree of alcohol, cleared with xylene, rapidly embedded in paraffin wax, and sectioned at 5 µm thick. The sections were stained with H&E and examined under light microscopy (Olympus CX23LEDRFS1, Olympus, Tokyo, Japan) to study the histopathological changes. Immunohistochemical staining was performed using TNF-α and caspase 3 rabbit polyclonal antibodies according to the manufacturer’s protocol. The positive control for TNF-α and caspase 3 was human tonsil, while for negative control slides, the same previous steps were carried out, but without the primary antibody addition (not included in results).

Morphometric estimation using Leica QWin 500 image analysis software (Leica Microsystems, Wetzlar, Germany) was performed for (a) semi-quantitation of histopathological changes and evaluation of spermatogenesis on 20 seminiferous tubules in different groups using Cosentino [52] and Johnsen [53] scores respectively, (b) assessment of the height of germinal lining epithelium using H&E-stained slides in 8 non-overlapping fields from each section of all rats of each group, and (c) evaluation of the mean area fraction of TNF-α and caspase 3 expression using immuno-stained slides in 8 non-overlapping fields for each group using ImageJ (freeware; rsbweb.nih.gov/ij).

### 4.6. Statistical Analysis

Results are represented as mean ± SEM. Data analysis was performed by a one-way analysis of variance (ANOVA) test followed by Tukey’s post-test for comparisons. The statistical analysis was done using GraphPad Prism software version 6.01 (San Diego, CA, USA) and *p* < 0.05 was considered significant.

## 5. Conclusions

Based on the current findings, it can be concluded that paeonol has a protective effect against MTX-mediated testicular injury. This protective effect is mediated via the mitigation of MTX-induced oxidative stress, inflammation, and apoptosis. Moreover, the upregulation of testicular P-gp expression observed in the current study presents a novel mechanism of paeonol-mediated gonadal protection.

## Figures and Tables

**Table 1:** Effect of paeonol on testicular weight, as well as serum testosterone and testicular P-glycoprotein levels.

Group	Testicular Weight (g)	Serum Testosterone (nmol/mL)	P-Glycoprotein (ng/mL)
**Control**	0.933 ± 0.07	2.93 ± 0.07	3.86 ± 0.32
**Paeonol**	0.969 ± 0.04	2.83 ± 0.09	3.70 ± 0.35
**MTX**	0.410 ± 0.08 *°	1.18 ± 0.09 *°	4.09 ± 0.39
**Paeonol + MTX**	0.738 ± 0.08 ^#^	2.16 ± 0.12 *°^#^	5.52 ± 0.43 *°^#^

Results are mean ± SEM (*n* = 6). *^,^°^,#^ Significantly different (*p* < 0.05) from control, paeonol, and MTX groups, respectively. MTX: methotrexate.

**Table 2:** Effect of paeonol on histopathological grades and spermatogenesis scoring.

Group	Histopathological Score	Height of Germinal Lining Epithelium (nm)	Spermatogenesis Score
**Control**	1.00 ± 0.00	145.0 ± 4.45	9.75 ± 0.16
**Paeonol**	1.00 ± 0.00	158.8 ± 2.70	9.87 ± 0.12
**MTX**	3.87 ± 0.12 *°	78.25 ± 2.59 *°	4.875 ± 0.35 *°
**Paeonol + MTX**	1.62 ± 0.26 *°^#^	137.3 ± 4.87 *°^#^	7.12 ± 0.39 *°^#^

Results are mean ± SEM (*n* = 8). *^,^°^,#^ Significantly different (*p* < 0.05) from control, paeonol, and MTX groups, respectively. MTX: methotrexate.

**Table 3:** Effect of paeonol on oxidative stress parameters.

Group	MDA (nmol/g Tissue)	NOx (nmol/g Tissue)	GSH (nmol/g Tissue)	SOD (U/g Tissue)
**Control**	14.30 ± 0.30	814.3 ± 66.57	309.9 ± 14.06	14019 ± 630.5
**Paeonol**	14.16 ± 0.37	807.5 ± 61.05	313.0 ± 12.42	14528 ± 546.4
**MTX**	45.63 ± 2.54 *°	2825 ± 225.1 *°	179.4 ± 10.67 *°	5814 ± 320.6 *°
**Paeonol + MTX**	16.23 ± 0.45 ^#^	968.4 ± 60.97 ^#^	262.1 ± 3.94 *°^#^	10579 ± 404.6 *°^#^

Results are mean ± SEM (*n* = 6). *^,^°^,#^ Significantly different (*p* < 0.05) from control, paeonol, and MTX groups, respectively. MTX: methotrexate, MDA: malondialdehyde, NOx: total nitrite, GSH: reduced glutathione, SOD: superoxide dismutase.
